# New ideas on how drivers perceive speed emerge from the fog

**DOI:** 10.7554/eLife.00281

**Published:** 2012-10-30

**Authors:** Jody C Culham

**Affiliations:** **Jody C Culham** is an *eLife* reviewing editor, and is in the Brain and Mind Institute, Department of Psychology, University of Western Ontario, London, Canada. jculham@uwo.ca

**Keywords:** motion perception, human psychophysics, virtual reality, driving simulation

## Abstract

Experiments with a driving simulator contradict previous results by showing that car drivers slow down in fog. However, other forms of reduced visibility can cause drivers to speed up.

**Related research article** Pretto P, Bresciani J-P, Rainer G, Bülthoff HH. 2012. Foggy perception slows us down. *eLife* **1**:e00031. doi: 10.7554/eLife.00031

**Image** Measuring motion perception in a driving simulator

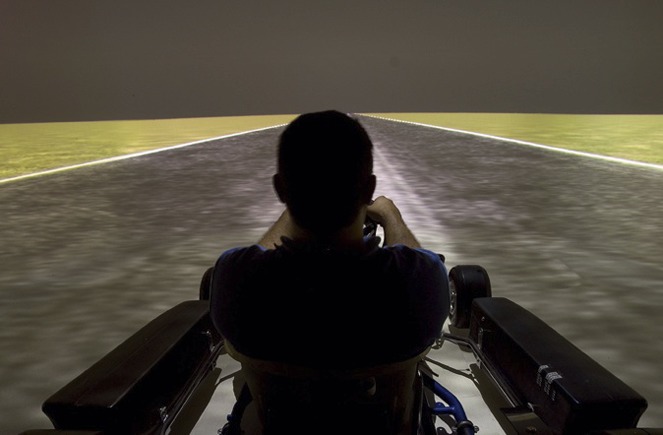

There is a stretch of Highway 401 in Canada that is known as ‘Carnage Alley’ because of the number of horrific accidents that have happened there. In fact, one of the worst accidents in Canadian history occurred in 1999 when 87 vehicles piled up after a thick blanket of fog descended on the highway. Statistics suggest that about a quarter of all car crashes are weather related, and that fog doubles the risk of an accident.

But what makes driving in fog so dangerous? One widely accepted explanation is that drivers underestimate their speed when driving in foggy conditions, so they speed up to compensate for this. However, in research published in *eLife*, Paolo Pretto, Jean-Pierre Bresciani, Gregor Rainer and Heinrich Bülthoff challenge this explanation with data from experiments in which state-of-the-art virtual-reality simulations are used to explore how drivers respond to conditions of reduced visibility (Pretto et al., 2012). The driving simulator used in the experiments is fitted with a panoramic virtual reality screen that fills the driver's entire field of view.

Past studies of speed perception simulated the effects of fog by reducing the contrast of everything in the scene equally regardless of distance (see, e.g., Snowden et al., 1998). Effectively, the simulation was more like driving while looking through a fogged up windshield than driving through actual fog.

Pretto et al.—who are based at the Max Planck Institute for Biological Cybernetics (PP and HHB), the University Pierre Mendès-France and the CNRS in Grenoble (J-PB) and the University of Fribourg (GR)—created a much more realistic simulation of fog: objects far away from the driver, such as the road surface close to the horizon, appeared fainter and fuzzier than nearby locations, such as the road surface right in front of the vehicle. In other words, the contrast was highest for objects nearest the driver and lowest for those further away. The experimenters used two levels of realistic fogginess (moderate and severe), and also two levels of uniform contrast reduction (again, moderate and severe) so that they could compare their results with previous studies.

In one experiment, 12 experienced drivers viewed two driving scenes that could differ in the visibility conditions and decided which of the two made them feel as if they were moving faster. In another experiment, 10 experienced drivers (none of whom had taken part in the first experiment) were trained to drive at a target speed during clear visibility based on feedback from a speedometer; then they attempted to match that speed under conditions of reduced visibility and without feedback.

**Figure 1:**
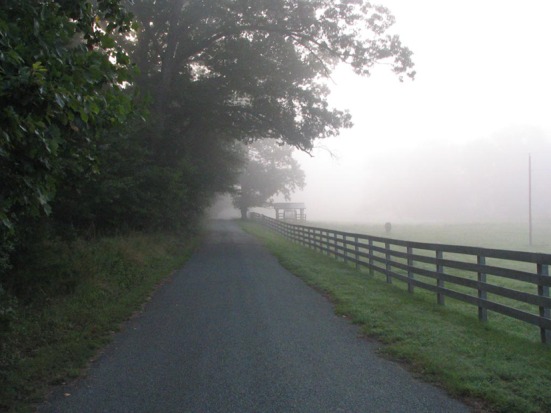
Fog doubles the risk of an car accident, which is why researchers are keen to understand how it influences how drivers perceive their speed.

Pretto et al. found that—contrary to previous results—the test drivers actually overestimated their speed during the natural fog simulations, and thus drove slower to compensate. Specifically, while drivers had an average speed of 85 km/hr under clear visibility, they decreased their speed to 77 and 71 km/hr for moderate and severe fog, respectively. Moreover, the authors replicated previous results, showing that a uniform reduction in contrast led drivers to underestimate their speed and thus speed up (to 101 km/hr for severe reductions).

Cleverly, they also created an ‘anti-fog’ simulation in which near objects had lower contrast than far objects. As with the uniform reductions in contrast, the anti-fog led drivers to underestimate their speed and to speed up dramatically (from 68 to 104 km/hr), which is the opposite of what happened for realistic fog.

Taken together, these results confirm that fog influences speed perception, and that this illusion causes drivers to slow down in real fog, as well they should. While many past studies, including studies of visual perception and the visual areas of the brain, have examined how changes in overall contrast affect perception, these results suggest that our visual system responds to the gradient of contrast differences rather than to overall levels of contrast.

Based on the finding that the drivers responded to fog and anti-fog in completely different ways, Pretto et al. suggest that one important factor may be the contrast gradient between central vision (where the participant is looking directly forward, down the road in this case) and peripheral vision (toward the edges of the scene, such as the roadsides in this case). This explanation is certainly plausible and parsimonious. However, it might be that speed perception is influenced by the way that contrast depends on distance from the driver. In the real world objects in the lower visual field (that is, objects below the viewer's line of sight, such the ground or the road) tend to be closer than objects in the upper visual field (such as clouds). Further experiments, perhaps even adding 3D vision to the displays, could disentangle the dependence of perceived speed on the contributions from various gradients (including central-peripheral, upper-lower, or near-far gradients).

Although this research eliminates one of the simplest and most intuitively appealing explanations for the increased likelihood of car accidents in fog, it could eventually lead to a better understanding of weather-related accidents. More importantly, with increasingly realistic simulations of real-world conditions in the safety of the laboratory environment, researchers can begin to search for possible technical solutions and driver strategies to help reduce accidents in the future.

A valuable next stage would be to make the simulations even more complex and naturalistic, adding additional features (such as hills, curves, lane boundaries and landmarks) to the scene. It would also be valuable to examine how the distance of perceived objects, particularly other vehicles, is affected by realistic fog conditions. Perhaps through such simulations, researchers can help drivers avoid devastating consequences in places such as Carnage Alley.
